# Urinary microbiota changes among NMIBC patients during BCG therapy: comparing BCG responders and non-responders

**DOI:** 10.3389/fcimb.2025.1479795

**Published:** 2025-03-10

**Authors:** Toni Boban, Blanka Milić Roje, Dora Knezović, Ana Jerončić, Hrvoje Šošić, Marijan Šitum, Janoš Terzić

**Affiliations:** ^1^ Department of Urology, University Hospital of Split, Split, Croatia; ^2^ Laboratory for Cancer Research, University of Split School of Medicine, Split, Croatia; ^3^ Department of Research in Biomedicine and Health, University of Split School of Medicine, Split, Croatia

**Keywords:** urinary microbiome, non-muscle invasive bladder cancer, BCG, response to therapy, immunotherapy

## Abstract

The gold standard for treating high-risk non-muscle-invasive bladder cancer involves the transurethral removal of cancerous tissue followed by BCG immunotherapy. So far, there is no reliable biomarker for predicting BCG efficacy and identifying patients who will or will not respond to BCG treatment. Emerging evidence suggests that urinary microbiota may play a crucial role in BCG efficacy. This study aimed to explore (i) changes in urinary microbiota during the six induction cycles of BCG and (ii) its potential predictive role in determining the outcome of BCG treatment. To this end, catheterized urine samples were collected before each of the six BCG doses and bacterial composition was analyzed using 16S rRNA gene sequencing. Patient inclusion criteria were male gender, no previous history of urothelial cancer, no other malignancies, no active infection, and no antibiotic usage for at least 20 days before the first BCG dose. We observed a significant decrease in biodiversity, measured by the Shannon Index, during the first week of therapy in 10 out of 12 patients (p=0.021). Additionally, differences in microbiota composition before the start of BCG therapy were noted between responders and non-responders to BCG therapy. Non-responders exhibited a 12 times higher abundance of genus *Aureispira* (p<0.001), and, at the species level, a 27-fold lower abundance of *Negativicoccus succinivorans* (p<0.001). Throughout the treatment, the abundance of the genus *Aureispira* decreased, showing an eightfold reduction by the end of therapy among non-responders (p<0.001). Our findings suggest that urinary microbiota plays an active role before and during the course of BCG therapy. However, this is a preliminary study, and further research involving larger patient cohorts is needed.

## 1 Introduction

According to the latest GLOBOCAN data, bladder cancer is the 10th most common cancer globally, with an estimated 549,000 new cases and 200,000 deaths yearly. Incidence among men is four times higher, ranking it the sixth most common cancer and the ninth leading cause of cancer death among men (Sung et al., 2021). In the USA, the 5-year average survival of bladder cancer patients is 77%, dropping to only 5% in those with metastatic disease (Saginala et al., 2020).

Bladder cancer is usually presented with hematuria whereas diagnosis is confirmed with a combination of cystoscopy and tumor biopsy. Non-muscle-invasive bladder cancer (NMIBC) accounts for 75% of bladder cancer pathology. The gold standard of care for high-risk NMIBC is a combination of transurethral resection of cancerous tissue (TURBT) with *Bacillus Calmette–Guerin* (BCG) adjuvant immunotherapy. Even though this therapy has a relatively high success rate, complete response is achieved in 55%-65% of papillary tumors and 75% of carcinoma *in situ* (CIS). However, around 40% will develop a recurrence despite initially responding to therapy (Lamm et al., 1991; Lamm, 1992; Lamm et al., 2000). This reveals a need to develop a biomarker that can stratify responders to BCG immunotherapy from non-responders. A significant portion of patients with NMIBC that do not respond to the standard therapy will progress to muscle-invasive bladder cancer (MIBC) and have a worse prognosis than patients with bladder cancers that were invasive at the time of diagnosis (Pietzak et al., 2019).

The discovery of the wide community of bacteria in urine samples, previously deemed sterile by traditional cultivation techniques, called “urinary microbiota” or “urobiota,” introduced a new research opportunity to understand bladder cancer pathogenesis and its response to therapy. A complex community of microorganisms that reside in the urinary bladder is thought to be responsible, through interaction with host immune cells and urothelial cells, for creating a microenvironment that could potentially promote or inhibit tumor development as well as modulate therapeutic outcomes.

Hence, our objective is to investigate alterations in the urobiota during BCG immunotherapy cycles. This exploration aims to provide a deeper understanding of the urobiota changes linked to a favorable response to the therapy, while also shedding light on the protective mechanisms underlying BCG immunotherapy in non-muscle-invasive bladder cancer.

## 2 Materials and methods

### 2.1 Patient recruitment

**Figure 1:**
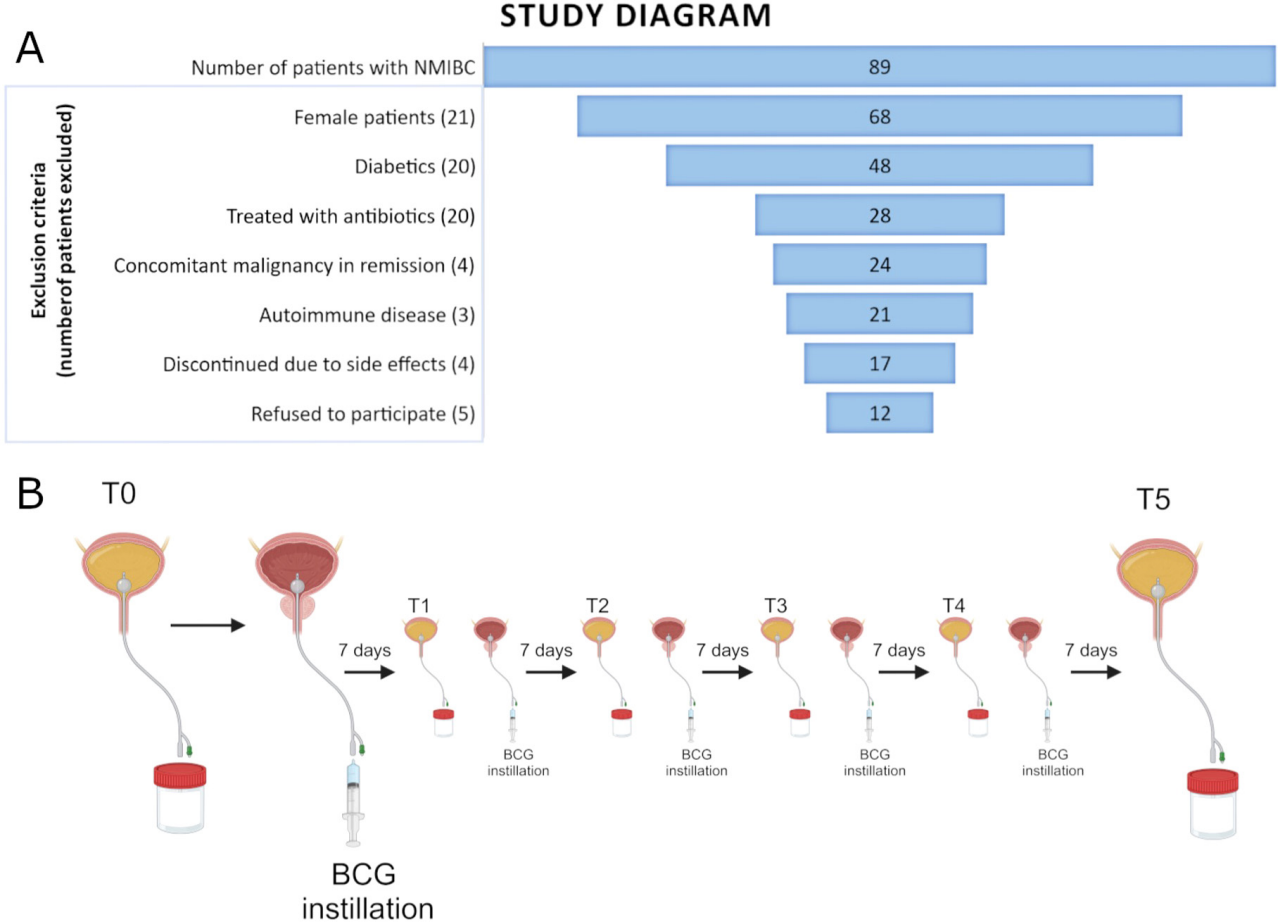
**(A)** Funnel plot of all patients with NMIBC recruited for the study and the exclusion criteria used for the selection of the final cohort. **(B)** Figure scheme of the sample collection of catheterized urine prior to each of the six induction BCG instillations.

This study was approved by the Board of Ethical Committee of Clinical Hospital Center Split (2181-147-01/06/M.S.-20-4). All patients have signed a written informed consent. A total of 12 male patients who underwent TURBT and were treated with adjuvant BCG immunotherapy for high-risk NMIBC were included in this study. Inclusion criteria for patient selection were male gender, no previous history of urothelial cancer, no other site malignancies, no active infection, and no antibiotic usage for 20 days prior to the first BCG dose application and during BCG cycles. Immunomodulating conditions such as autoimmune diseases and diabetes were considered as exclusion criteria since they can modulate urobiota composition (Shaheen et al., 2022). Also, all patients were preoperatively treated with the same prophylaxis (norfloxacin). The median follow-up for the whole cohort is 38.5 months. The recurrence was diagnosed with a mean follow-up of 14 months. One patient’s follow-up was limited to 10 months due to a death unrelated to bladder cancer and was excluded from the analysis of response-to-therapy groups, but remained included in all other analyses (labeled Limited FU). The diagram of patient selection with the exclusion process is presented in **Figure 1A**, whereas **Table 1** shows the patient’s demographics and relevant clinical data.

**Table 1:** Patients’ clinical characteristics.

Number of patients	12
Gender	Male 12
	Female 0
Age (median)	72
Smoking status	Yes 4 (42%)
	No 7 (58%)
T stage	TaLG (8%)
	CIS 3 (25%)
	T1HG 5 (42%)
	T1HG + CIS 3 (25%)
Antibiotics <20 days prior to BCG	Yes 0
	No 12 (100%)
Follow-up (months)	38.5
Recurrence (high-grade)	Yes 3 (25%)
	No 9 (75%)
History of high-grade UC	Yes 0
	No 12 (100%)

### 2.2 Urine collection

Urine samples were obtained with aseptic bladder catheterization before each of the six induction BCG doses. Therefore, the first sample (timepoint T0) represents the patient’s urinary microbiota prior to BCG instillation, whereas all other samples (timepoints T1-T5) represent the urobiota composition during the course of the BCG induction therapy (shown in **Figure 1B**). Urine samples were stored in a sterile container and were transported to the Laboratory for Cancer Research at the University of Split, School of Medicine, within 4 h for further processing.

### 2.3 DNA isolation and 16S rRNA gene sequencing

Approximately 30 to 50 mL of collected urine was centrifuged for 10 min at 7,500 g. The supernatant was discarded, and pellets were resuspended in 100 µL of molecular-grade water and stored at −20°C. Alternatively, whole urine samples were frozen at −80°C, and centrifugation was performed after thawing.

DNA isolation was performed using urine pellets as described previously (Bučević Popović et al., 2018). We used a DNA PowerSoil Kit (Qiagen, Germany) according to the manufacturer’s protocol. Extracted DNA was eluted in 20 µL of water and stored at −20°C. DNA concentrations were determined by NanoDrop (Thermo Fisher, USA) and Qubit fluorometric assay (Thermo Fisher, USA) and ranged from too low for detection to 500 ng/µL. To check for DNA contamination during DNA extraction and sample collection, we performed negative controls using DNA-free molecular-grade water that was passed through the sterilized catheter, followed by the same DNA extraction procedure. Negative controls did not give DNA bands in the PCR reactions.

The PCR reaction was performed using the universal primers 515F (5′-GTGCCAGCMGCCGCGGTAA-3′) and 806R (5′-GGACTACHVGGGTWTCTAAT-3′) that amplify the hypervariable region 4 (V4) of the bacterial 16S ribosomal gene (Caporaso et al., 2011; Caporaso et al., 2012). The second PCR step was performed to add Illumina adapters and sample indices. The same amount of PCR products from each sample was pooled; the mix was purified and then size selected using Agencourt AMPure XP-PCR magnetic beads (Beckman Coulter, USA).

Sequencing was performed using a paired-end 2 bp × 250 bp sequencing reagent cartridge, according to manufacturer’s protocol instructions (Illumina, USA). The library was quantified with Qubit and real-time PCR and checked with a bioanalyzer for size distribution detection. PCR and sequencing were performed at Novogene Company (Novogene Co., China).

### 2.4 Bioinformatic analysis

Paired-end reads were assigned to samples according to their unique indices and truncated by cutting off the barcode and primer sequences. Demultiplexed reads were imported into the Qiime2 platform (McKinney, 2010; McDonald et al., 2012; Bolyen et al., 2019). Trimmed reads were denoised using the DADA2 (2023.5.0) package using standard parameters (Callahan et al., 2016). This included filtering out of low-quality, PhiX, and chimera reads and finally generation of amplicon sequence variants (ASVs). Next, we used a classifier based on the SILVA database (v.138) to assign taxonomy to the sequences in the ASV table (Pruesse et al., 2007; Quast et al., 2013; Yilmaz et al., 2014). Naïve Bayes pretrained classifier Silva 138 99% OTUs using the 515F/806R region of sequences from Qiime2 were used for this purpose (Bokulich et al., 2018; Robeson et al., 2021).

Microbiome composition was investigated using species relative abundance and alpha and beta diversity indices (Pielou, 1966; Vázquez-Baeza et al., 2013; Weiss et al., 2017). Phylogenetic analysis was performed using MAFFT multiple-sequence alignment and FastTree phylogenetic tree building (Price et al., 2010; Katoh and Standley, 2013).

Data analysis in R using RStudio was performed using packages mia and vegan for microbiome analysis (Oksanen et al., 2012; Ernst et al., 2024). To make graphical representations, package ggplot2 was used (Wickham, 2016). Differential abundance testing was performed in R version 4.2.2 using the ANCOMBC-2 and Linda packages on phylum, family, and genus taxonomic levels (Lin and Peddada, 2020; Zhou et al., 2022). Paired-sample differential abundance was performed using the ALDEx2 package in R (Gloor et al., 2016; Wickham et al., 2019). Alpha diversity (Shannon index, species richness) and beta diversity (Bray–Curtis) indices were calculated using a rarefied table with the same number of reads for all samples (Gloor et al., 2016,241). This depth was chosen according to lowest read number per sample in order to keep all samples in the analysis. From rarefaction curves of alpha diversity measures, this depth was estimated as satisfactory (**Supplementary Figure 1**).

A total of 5,596,063 reads were obtained from 72 urine samples (mean frequency of reads per sample: 77,723, range: 30,241-101,073). Sequences were assigned to 42,212 amplicon sequence variants or ASVs (mean frequency of reads per ASV: 132.57, range: 1-870,084).

### 2.5 Statistical analysis

We performed data analysis using MedCalc statistical software (version 23.0.6 MedCalc Software Ltd., Ostend, Belgium).

The distribution of Shannon’s Biodiversity Index (SI) as well as ASV at different time points was summarized with the median and range due to the limited sample size, and the associated 95% confidence intervals for these changes were also reported.

To test for significant differences in both Shannon’s Index and ASV across different time points, we used the Friedman test, with the Wilcoxon test for paired samples applied as *post hoc* analysis. The signed rank sum test was additionally used to determine whether the median of changes relative to baseline differed from zero. The association between weekly change in SI and the initial biodiversity level of an individual was investigated using simple linear regression. The two-sided significance level was set at 0.05.

## 3 Results

### 3.1 Bacterial composition

**Figure 2:**
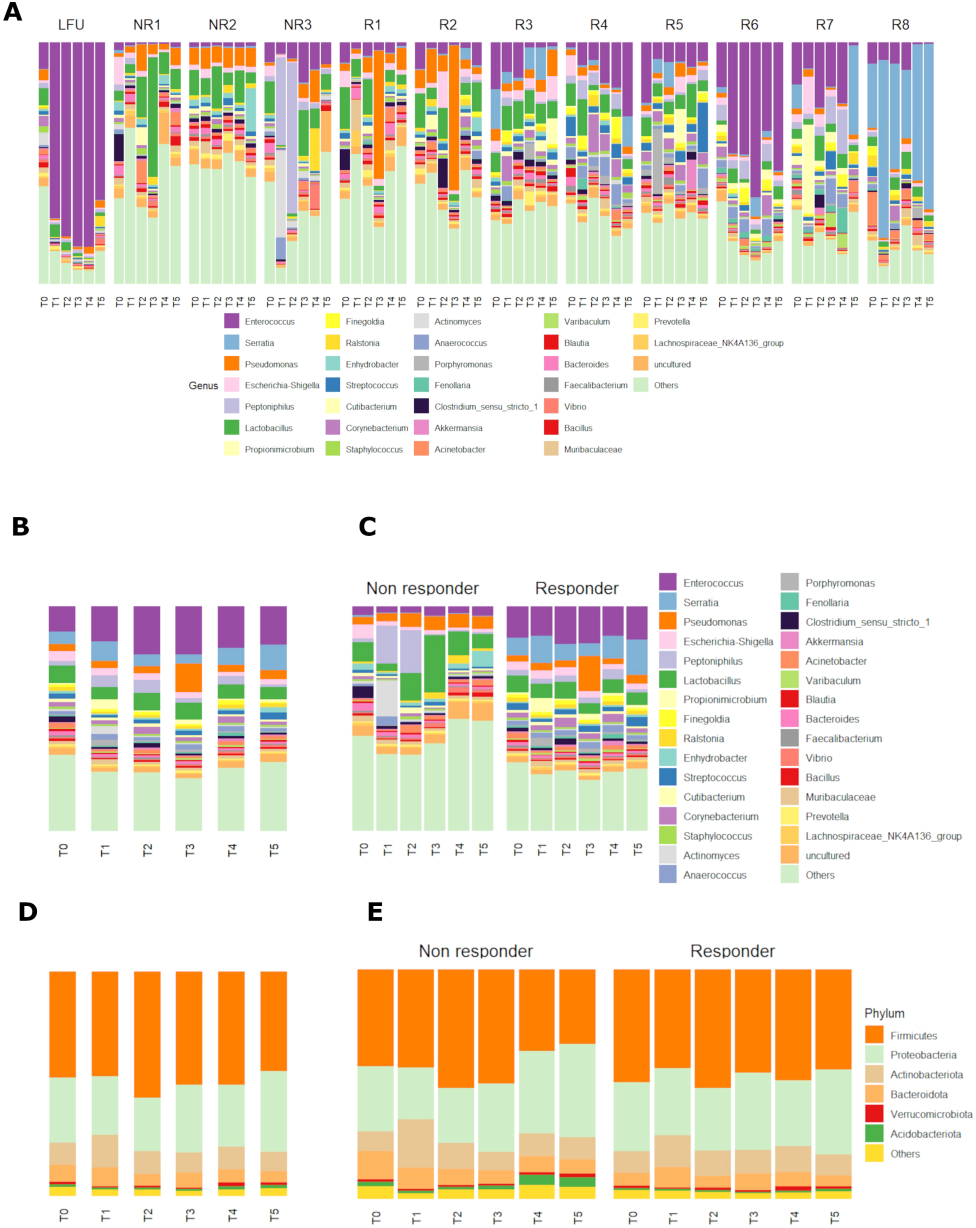
**(A, B)** Relative abundance of bacterial genera in all patients individually **(A)** and grouped **(B)** in six different timepoints before and during the BCG induction therapy. **(C)** Relative abundance of bacterial genera in patients grouped by response to therapy and shown at six different timepoints in the BCG induction therapy. **(D, E)** Relative abundance of bacterial phyla in all patients **(D)** and patients grouped by response to therapy **(E)** and shown at six different timepoints in the BCG induction therapy. (T0-T5—therapy timepoints, R—responders to therapy, NR—non-responders to therapy, LFU—patient with limited 10-month follow-up of no cancer recurrence).

The relative abundance of the most abundant genera and phyla of bacteria recorded in all patients at all timepoints is shown in **Figure 2A**. *Enterococcus* sp. (17.6%), *Serratia* (7.1%), *Pseudomonas* (4.9%), and *Escherichia–Shigella* (2.8%) were overall the most prevalent genera (**Figures 2A–C**). Firmicutes (49.1%) and Proteobacteria (28.7%) were overall the most prevalent phyla, followed by Actinobacteriota (10.4%) and Bacteriodota (6.3%) (**Figure 2D**).

When looking at the relative composition in urinary microbiota before the BCG therapy (timepoint T0), the most prevalent genera for all patients were *Enterococcus* sp. (11.35%), *Lactobacillus* sp. (7.83%), *Serratia* sp. (5.64%), and *Escherichia–Shigella* sp. (4.14%) (**Figure 2B**).

There were differences in most abundant taxa in the two response-to-therapy groups when looking at all timepoints. *Enterococcus* (14.7%), *Serratia* (9.8%), and *Lactobacillus* (6.3%) were the most prevalent genera in the responder group (**Figure 2C**). In the non-responder group, most prevalent genera were *Lactobacillus* (10.3%), *Peptoniphilus* (7.0%), and *Pseudomonas* (5.0%). The most prevalent phyla (**Figure 2E**) were similar in both response-to-therapy groups, the most abundant being Firmicutes in the responder group (46.7%) and in the non-responder group (42.2%), followed by Proteobacteria (responder—30.9%, non-responder—29.8%) and Actinobacteriota (responder—10.9%, non-responder—11.9%).

Before the first BCG administration (timepoint T0), the most prevalent genera in responders were *Enterococcus* (13.9%), *Serratia* (7.8%), and *Lactobacillus* (7.6%), whereas in non-responders, *Lactobacillus* (8.7%), *Escherichia–Shigella* (6.2%), and *Clostridium* (5.2%) (**Figure 2D**). Most abundant phyla before the first BCG administration were similar in both response groups: Firmicutes (R—49,2%, NR—42.2%), Proteobacteria (R—29,9%, NR—28.3%), Actinobacteriota (R—9.4%, NR—8.8%), and Bacteroidota (R—5.7%, NR—12.1%) (**Figure 2E**).

In the last timepoint (T5—before the last induction BCG dose), the three most abundant genera were the same as in T0 for BCG responders: *Serratia* (15.8%), *Enterococcus* (14.8%), and *Lactobacillus* (5.4%), whereas for non-responders, the three most abundant genera changed to *Enhydrobacter* (6.9%), *Lactobacillus* (6.5%), and *Pseudomonas* (5.4%) (**Figure 2D**). The most abundant phyla in the last timepoint were Firmicutes (R—43.7%, NR—32.6%), Proteobacteria (R—36.9%, NR—40.5%), Actinobacteriota (R—9.2%, NR—9.8%), and Bacteroidota (R—4.8%, NR—6.1%) (**Figure 2E**).

### 3.2 Bacterial diversity measures

**Figure 3:**
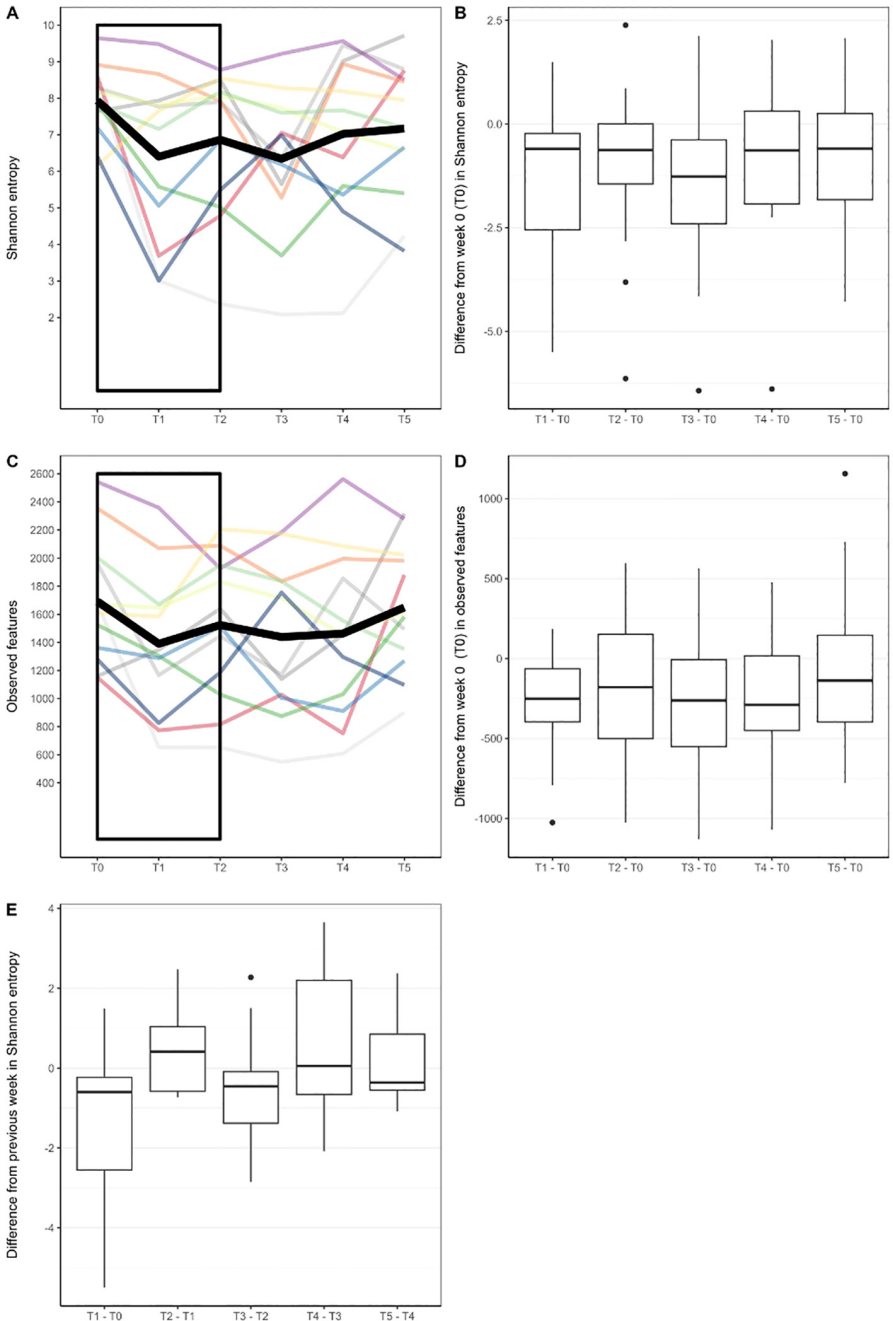
**(A)** Biodiversity as measured with Shannon Index in different timepoints. Each line represents alpha diversity volatility throughout six timepoints for one patient. Thick black line represents mean values. **(B)** Difference in Shannon Index from baseline value (week 0) in later timepoints. **(C)** Number of observed bacterial features (measure of species richness) in different timepoints. **(D)** Difference in observed bacterial features from baseline value (week 0) in later timepoints. **(E)** Difference in Shannon entropy from a previous week. Rectangles in **(A, C)** were added to highlight where significant changes in biodiversity measures were found.

The baseline Shannon’s Biodiversity Index (SI) for most respondents (50%) was between 7.5 and 8.5 (median SI 8.0, range 6.2-9.6) (**Figure 3A**). We observed a significant change in biodiversity, as measured by the SI, across the six therapy time points (Friedman test, p=0.037). Specifically, there was a notable decrease in the SI compared with the baseline level after the first and third weeks of therapy (p ≤ 0.041), whereas during the other therapy weeks (weeks 2, 4, and 5), the SI showed no significant deviation from the baseline (**Figures 3A, B**). In the initial week of therapy, following the application of BCG, a decrease in biodiversity was noted in 10 out of 12 patients (signed rank sum test, p=0.021), with a median SI decrease of 1.3 (95% CI 0.2, 2.9).

Regarding the species, more precisely ASVs, richness of the bacterial community, we also observed significant changes in the number of observed features relative to the baseline value at the 0.1 significance level (Friedman test, p=0.081). The number of bacterial community features was notably lower only during the first week of therapy (p=0.005), returning to levels comparable with baseline thereafter (**Figures 3C, D**).

When assessing weekly biodiversity changes (between successive therapy doses), we made an interesting observation. In the second week of treatment (T2), nearly three-quarters of patients (8 out of 12 subjects) exhibited an increase in SI compared with their SI levels in the first week of therapy (T1) (**Figure 3E**). However, the median increase did not reach statistical significance (signed rank sum test, p=0.176). In the subsequent weeks of therapy, microbial biodiversity in response to therapy was not so pronounced as we did not observe significance in the direction of weekly changes in terms of growth or decline (signed rank test, p≥0.204), nor did we find a significant correlation between weekly changes and the initial state of biodiversity of each individual (R2 ≤ 13%, p-value for regression coefficient ≥0.244).

### 3.3 Response to therapy and bacterial diversity measures

When considering alpha diversity variance attributed to response-to-therapy status, responders had non-significantly lower mean Shannon diversity before the first BCG instillation, (median(R) = 7.40, median (NR) = 8.55, p-value = 0.29, Wilcoxon test with Holm adjustment, **Supplementary Figure 2A**). Species richness was similar in responders and non-responders in the first time point (median (R)= 1582.5, median (NR) = 1674.5, p-value = 1, Wilcoxon test with Holm adjustment, **Supplementary Figure 2B**). Overall microbial community dissimilarity, calculated as Bray–Curtis dissimilarity, did not show any differentiation based on response status (p-value 1, Permanova test, stratified per patient, number of permutations 9999, **Supplementary Figure 3A**); however, when comparing only the urobiome community before the start of the therapy (T0), the differentiation was significant at level 0.1 (p-value 0.0539, Permanova test, number of permutations 9999) (**Supplementary Figure 3B**), whereas in the last timepoint (T5), no significant clustering was detected (p-value 0.1856, Permanova test, number of permutations 9999, **Supplementary Figure 3C**).

### 3.4 Differential abundance testing

**Figure 4:**
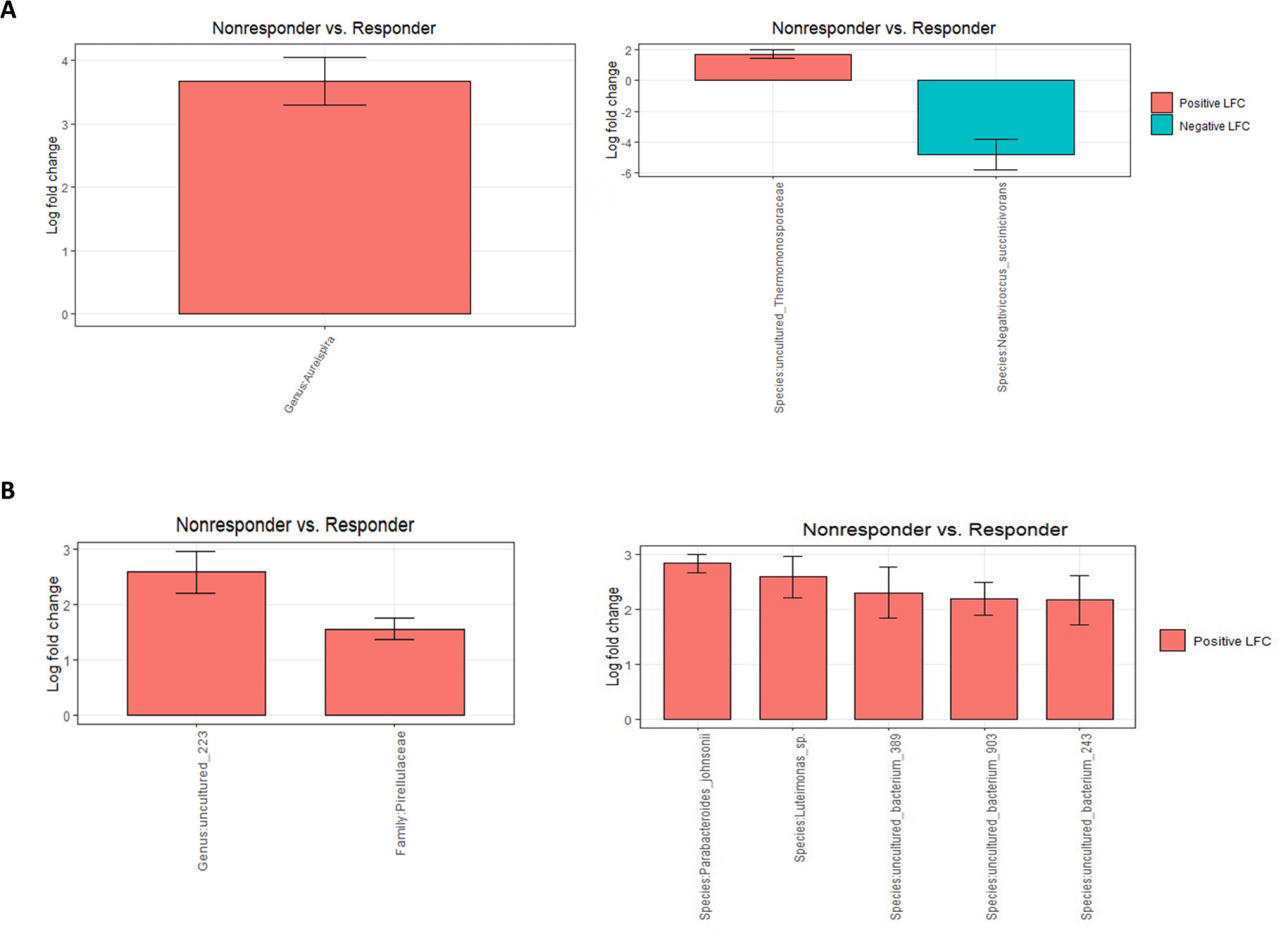
Differential abundance analysis (ANCOMBC2) of urine samples between patients grouped by response to therapy: **(A)** before the onset of the therapy (T0) at genus and species levels and **(B)** at the end of the therapy (T5) at genus and species levels. The values shown in the figure are expressed as the logarithm base 2 of the fold change value (Log_2_FC). The results are represented as either positive or negative, depending on whether there is an increase or decrease in the abundance, respectively. LFC—logarithm base 2 of the fold change value.

Differential abundance analysis revealed that before the onset of the BCG treatment, non-responders had 12 times higher abundance of genus *Aureispira* compared with ones that did respond to treatment (p < 0,001) (**Figure 4A**). On a species level*, Negativicoccus succinicivorans* showed 27 times lower abundance among non-responders (p < 0,001) (**Figure 4A**). At the end of the BCG treatment (**Figure 4B**), genera and species related to the phylum *Pirellulaceae* had three (p < 0,001) times higher abundance in the non-responder’s group, along with species the *Parabacteroides johnsonii*, showing the highest increase in abundance (p < 0,001), almost three times.

**Figure 5:**
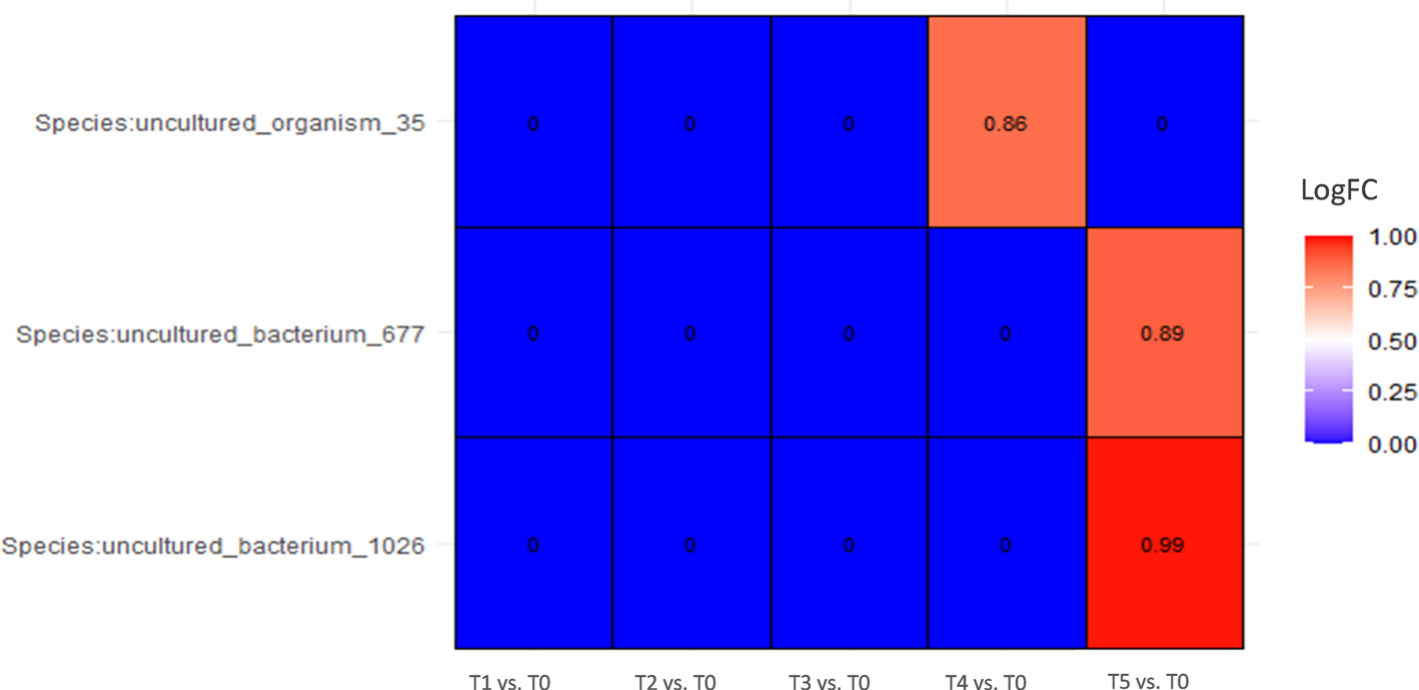
Differential abundance analysis of urine samples between therapy timepoints in patients who responded to therapy (responders) on species level (ANCOMBC2 analysis). The values shown in the figure are expressed as the logarithm base 2 of the fold change value (Log_2_FC). The results are represented as either positive or negative, depending on whether there is an increase or decrease in the abundance, respectively, relative to the starting point before the onset of the therapy (T0). LogFC—logarithm base 2 of the fold change value.

In patients that responded to the BCG treatment, the only difference during the course of treatment was observed at the final stage of therapy, showing an increase in abundance of almost two times in several species (p < 0,001, ANCOMBC2) compared with a starting point, but all of them were uncultured (**Figure 5**).

**Figure 6:**
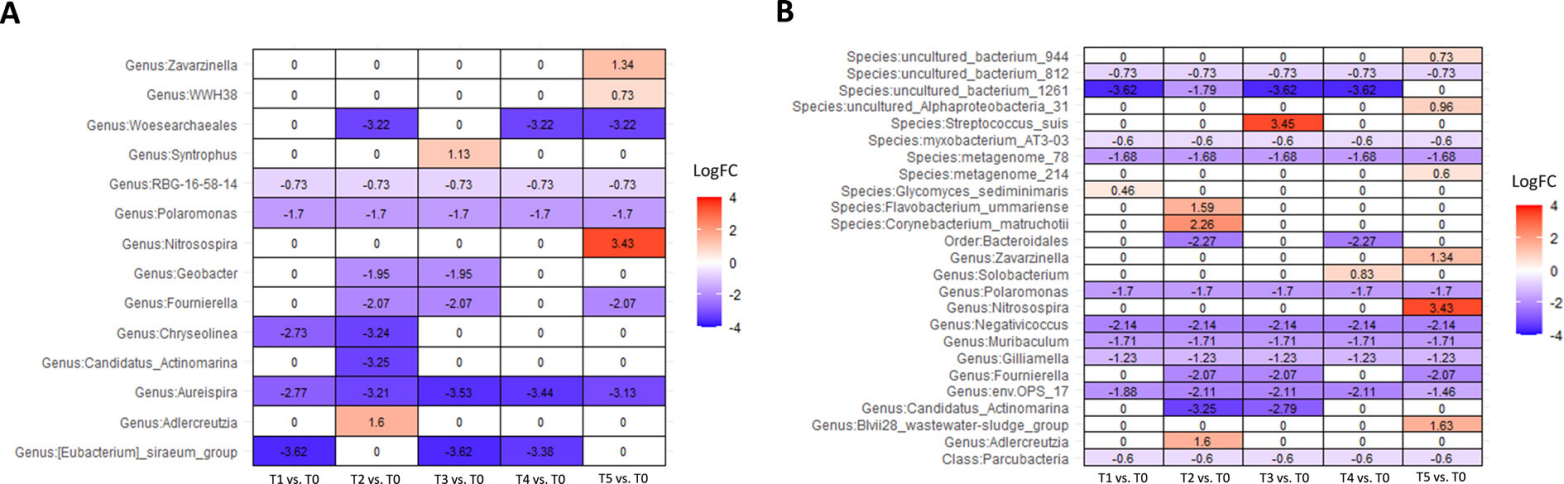
Differential abundance analysis of urine samples between therapy timepoints in patients who did not respond to therapy (non-responders) on **(A)** genus and **(B)** species levels (ANCOMBC2 analysis). The values shown in the figure are expressed as the logarithm base 2 of the fold change value (Log_2_FC). The results are represented as either positive or negative, depending on whether there is an increase or decrease in the abundance, respectively, relative to the starting point before the onset of the therapy (T0). LogFC—logarithm base 2 of the fold change value.

In a group of patients that did not respond to therapy, Linda analysis revealed a significant decline in the abundance of phylum Nanoarchaeota, up to 60 times (p = 0,025) after the first administration of the BCG treatment and an additional decline to 135 times by the end of the therapy (p = 0.005) (**Supplementary Figure 4**). **Figure 6** shows the dynamics of abundance changes on a genus and species levels throughout the course of the therapy in the non-responder group (ANCOMBC2 analysis). Genus *Nitrosospira* had 10 times higher abundance at the end of the BCG treatment compared with the starting point (p < 0.001). Furthermore, genus *Aureispira*, except for being more abundant in non-responders before the onset of the therapy, also showed a decreasing trend in abundance throughout the treatment period among non-responders. At the end of the BCG therapy, genus *Aureispira* was eight times less abundant (p < 0.001) compared with the values before the onset of the treatment.

Furthermore, we did pair sample testing on samples from the same patient to detect differences in taxon abundance between two therapy timepoints (T0 vs. T1, T1 vs. T2, and T0 vs. T5) at levels of species, genus, family, and phylum, but no taxons were detected to be significantly changed.

Regarding BCG bacterial detection, we were not able to determine up to species-level sequences belonging to the genus *Mycobacterium*. This genus represented 0.18% sequences in both responders and non-responders before the first instillation (T0). In the last timepoint, its relative abundance in responders was higher, 0.33%, whereas in non-responders it was somewhat lower at 0.14% (**Supplementary Figure 5**).

## 4 Discussion

In our study, we found differences in urinary microbiota composition between responders and non-responders, as well as a decrease in microbial biodiversity in both patient groups following the administration of BCG. Although BCG immunotherapy is a well-established therapy for NMIBC, it is unknown how it impacts the bladder microbiome and how urinary microbiota modulates the BCG’s efficacy (Herr and Morales, 2008). Thus, this research aimed to describe microbiota composition before BCG therapy, and possibly find microbial predictors of the patient’s positive response to BCG. Additionally, we wanted to gain insight into the dynamics of microbiota change through the course of BCG therapy and to get additional insight into the mechanism of BCG antitumorigenic action (Redelman-Sidi et al., 2014). This is the first study that follows urinary microbiota changes during the full induction course of BCG treatment.

The most prevalent genera among responders before the start of BCG therapy (T0 time point) were *Enterococcus*, *Serratia*, and *Lactobacillus*, whereas in non-responders *Lactobacillus*, *Escherichia*–*Shigella*, and *Clostridia* prevailed. Despite differences in prevalence, no statistical significance was found, similar to the lack of difference between NMIBC and control individuals described previously (Heidrich et al., 2024). However, Knorr et al. reported differences among genera between responder and non-responder patients (Knorr et al., 2024). When all bacteria were analyzed at a genus level, *Aureispira*, a Gram-negative aerobic genus, was found to be more abundant among our non-responder group (Yuasa et al., 2023). This genus was not previously reported to be part of the urinary microbiota; however, its family, *Saprospiraceae*, has been previously linked to the urinary microbiota of women with interstitial cystitis (Siddiqui et al., 2012).

Similarly to *Aureispira*, an uncultured species in the family of aerobic, Gram-positive *Thermomonosporaceae* family was also found to be more abundant in non-responders. On the contrary, species *Negativicoccus succinicivorans* was found to be 27 times more abundant among the patients that responded to the BCG therapy. *Negativicoccus succinicivorans* is a little-known Gram-negative anaerobic bacterium, isolated from clinical specimens (Marchandin et al., 2010). It has also been reported to be part of the healthy male and female urinary microbiome and less abundant in the urine of patients with type 2 diabetes compared with healthy controls (Calvigioni et al., 2024). Since there is currently limited research on the microbes distinguishing responders from non-responders, their significance in BCG therapy remains largely speculative. Nonetheless, if these distinctions persist in subsequent studies, these microbes could serve as biomarkers to predict responsiveness to BCG therapy. Identifying such biomarkers could spare patients who would not benefit from the treatment, offering a more targeted approach.

We observed biodiversity changes throughout the course of BCG therapy. The most significant was the reduction of biodiversity after the initial dose of BCG observed among responders and non-responders. A somewhat similar decrease in bacterial richness was noted previously (James et al., 2023), indicating an active role of BCG competing for space, nutrients or releasing molecules affecting other community members. This initial drop in diversity is mostly diminished during the continuation of therapy. However, at the end of the urine collection, 5 weeks from the first dose of BCG, urobiota composition slightly changed. For example, family *Pirellulaceae*, species *Parabacteroides Johnsonii*, *Luteimonas* sp., and some uncultured bacteria were more prevalent among non-responders.

In conclusion, our study offers preliminary insights into urinary microbiota changes during BCG therapy and its relation to the response of this treatment. Differences in abundances in taxa *Aureispira* and *Negativicoccus succinicivorans* suggest a difference in urobiota composition of responders and non-responders. The found decrease in biodiversity richness following the start of BCG, as well as differences in taxon abundance in the final time point suggest an active interplay between instilled BCG and the local urobiota and active role of urinary microbiota in BCG effectiveness. However, further investigations involving a larger, well-stratified patient population are warranted to validate these findings conclusively.

## Data Availability

The datasets presented in this study can be found in online repositories. The names of the repository/repositories and accession number(s) can be found below: https://www.ncbi.nlm.nih.gov/, PRJNA1066269.

## References

[B1] Bokulich N. A. Kaehler B. D. Rideout J. R. Dillon M. Boylen E. Knight R.. (2018). Optimizing taxonomic classification of marker-gene amplicon sequences with QIIME 2’s q2-feature-classifier plugin. Microbiome 6, 90. doi: 10.1186/s40168-018-0470-z 29773078 PMC5956843

[B2] Bolyen E. Rideout J. R. Dillon M. R. Bokulich N. A. Abnet C. C. Al-Ghalith G. A.. (2019). Reproducible, interactive, scalable and extensible microbiome data science using QIIME 2. Nat. Biotechnol. 37 (8), 852–857. doi: 10.1038/s41587-019-0209-9 31341288 PMC7015180

[B3] Bučević Popović V. Šitum M. Chow C. T. Chan L. S. Roje B. Terzić J. (2018). The urinary microbiome associated with bladder cancer. Sci. Rep. 8, 12157. doi: 10.1038/s41598-018-29054-w 30108246 PMC6092344

[B4] Callahan B. J. McMurdie P. J. Rosen M. J. Han A. W. Johnson A. J. Holmes S. P. (2016). DADA2: High-resolution sample inference from Illumina amplicon data. Nat. Methods 13, 581–583. doi: 10.1038/nmeth.3869 27214047 PMC4927377

[B5] Calvigioni M. Biancalana E. Mazzantini D. Celandroni F. Rossi C. Mengozzi A.. (2024). In-depth microbiological characterization of urine from subjects with type 2 diabetes. J. Clin. Endocrinol. Metab. 110, 185–194. doi: 10.1210/clinem/dgae389 38870276 PMC11651686

[B6] Caporaso J. G. Lauber C. L. Walters W. A. Berg-Lyons D. Huntley J. Fierer N.. (2012). Ultra-high-throughput microbial community analysis on the Illumina HiSeq and MiSeq platforms. ISME J. 6, 1621–1624. doi: 10.1038/ismej.2012.8 22402401 PMC3400413

[B7] Caporaso J. G. Lauber C. L. Walters W. A. Berg-Lyons D. Lozupone C. A. Turnbaugh P. J.. (2011). Global patterns of 16S rRNA diversity at a depth of millions of sequences per sample. Proc. Natl. Acad. Sci. U S A. 108 Suppl 1, 4516–4522. doi: 10.1073/pnas.1000080107 20534432 PMC3063599

[B8] Ernst F. G. M. Shetty S. A. Borman T. Lahti L. (2024). mia: microbiome analysis. Available online at: https://github.com/microbiome/mia (Accessed January 27, 2024).

[B9] Gloor G. B. Macklaim J. M. Fernandes A. D. (2016). Displaying variation in large datasets: plotting a visual summary of effect sizes. J. Comput. Graphical Stat 25, 971–979. doi: 10.1080/10618600.2015.1131161

[B10] Heidrich V. Mariotti A. C. H. Inoue L. T. Coser E. M. Dos Santos E. X. Dos Santos H. D. B.. (2024). The bladder microbiota is not significantly altered by intravesical BCG therapy. Urol Oncol. 42, 22.e13-22.e21. . doi: 10.1016/j.urolonc.2023.11.003 38030469

[B11] Herr H. W. Morales A. (2008). History of bacillus Calmette-Guerin and bladder cancer: an immunotherapy success story. J. Urol. 179, 53–56. doi: 10.1016/j.juro.2007.08.122 17997439

[B12] James C. Gomez K. Desai S. Patel H. D. Rac G. Doshi C. P.. (2023). Impact of intravesical Bacillus Calmette-Guérin and chemotherapy on the bladder microbiome in patients with non-muscle invasive bladder cancer. Front. Cell Infect. Microbiol. 13:1125809. doi: 10.3389/fcimb.2023.1125809 37091677 PMC10114608

[B13] Katoh K. Standley D. M. (2013). MAFFT multiple sequence alignment software version 7: improvements in performance and usability. Mol. Biol. Evol. 30, 772–780. doi: 10.1093/molbev/mst010 23329690 PMC3603318

[B14] Knorr J. Lone Z. Werneburg G. Adler A. Agudelo J. Suryavanshi M.. (2024). An exploratory study investigating the impact of the bladder tumor microbiome on Bacillus Calmette Guerin (BCG) response in non-muscle invasive bladder cancer. Urol Oncol. 42, 291.e1-291.e11. doi: 10.1016/j.urolonc.2024.04.011 38664180

[B15] Lamm D. L. (1992). Long-term results of intravesical therapy for superficial bladder cancer. Urologic Clinics North America. 19, 573–580. doi: 10.1016/S0094-0143(21)00424-9 1636241

[B16] Lamm D. L. Blumenstein B. A. Crawford E. D. Montie J. E. Scardino P. Grossman H. B.. (1991). A randomized trial of intravesical doxorubicin and immunotherapy with bacille Calmette-Guérin for transitional-cell carcinoma of the bladder. N Engl. J. Med. 325, 1205–1209. doi: 10.1056/NEJM199110243251703 1922207

[B17] Lamm D. L. Blumenstein B. A. Crissman J. D. Montie J. E. Gottesman J. E. Lowe B. A.. (2000). Maintenance bacillus Calmette-Guerin immunotherapy for recurrent TA, T1 and carcinoma in *situ* transitional cell carcinoma of the bladder: a randomized Southwest Oncology Group Study. J. Urol. 163, 1124–1129. doi: 10.1016/S0022-5347(05)67707-5 10737480

[B18] Lin H. Peddada S. D. (2020). Analysis of compositions of microbiomes with bias correction. Nat. Commun. 11, 3514. doi: 10.1038/s41467-020-17041-7 32665548 PMC7360769

[B19] Marchandin H. Teyssier C. Campos J. Jean-Pierre H. Roger F. Gay B.. (2010). Negativicoccus succinicivorans gen. nov., sp. nov., isolated from human clinical samples, emended description of the family Veillonellaceae and description of Negativicutes classis nov., Selenomonadales ord. nov. and Acidaminococcaceae fam. nov. in the bacterial phylum Firmicutes. Int. J. Syst. Evol. Microbiol. 60, 1271–1279. doi: 10.1099/ijs.0.013102-0 19667386

[B20] McDonald D. Clemente J. C. Kuczynski J. Rideout J. R. Stombaugh J. Wendel D.. (2012). The Biological Observation Matrix (BIOM) format or: how I learned to stop worrying and love the ome-ome. Gigascience 1, 7. doi: 10.1186/2047-217X-1-7 23587224 PMC3626512

[B21] McKinney W. (2010). “Data structures for statistical computing in python,” in Proceedings of the 9th Python in Science Conference, Austin, 28 June-3 July 2010. (Austin, Texas: SciPy), 56–61. doi: 10.25080/Majora-92bf1922-00a

[B22] Oksanen J. Blanchet F. G. Kindt R. Legendre P. Minchin P. R. O’Hara R. B.. (2012). vegan: Community Ecology Package. (Vienna, Austria: CRAN (Comprehensive R Archive Network)).

[B23] Pielou E. C. (1966). The measurement of diversity in different types of biological collections. J. Theor. Biol. 13, 131–144. doi: 10.1016/0022-5193(66)90013-0

[B24] Pietzak E. J. Zabor E. C. Bagrodia A. Armenia J. Hu W. Zehir A.. (2019). Genomic differences between “Primary” and “Secondary” Muscle-invasive bladder cancer as a basis for disparate outcomes to cisplatin-based neoadjuvant chemotherapy HHS public access. Eur. Urol. 75, 231–239. doi: 10.1016/j.eururo.2018.09.002 30290956 PMC6339572

[B25] Price M. N. Dehal P. S. Arkin A. P. (2010). FastTree 2–approximately maximum-likelihood trees for large alignments. PloS One 5, e9490. doi: 10.1371/journal.pone.0009490 20224823 PMC2835736

[B26] Pruesse E. Quast C. Knittel K. Fuchs B. M. Ludwig W. Peplies J.. (2007). SILVA: a comprehensive online resource for quality checked and aligned ribosomal RNA sequence data compatible with ARB. Nucleic Acids Res. 35, 7188–7196. doi: 10.1093/nar/gkm864 17947321 PMC2175337

[B27] Quast C. Pruesse E. Yilmaz P. Gerken J. Schweer T. Yarza P.. (2013). The SILVA ribosomal RNA gene database project: improved data processing and web-based tools. Nucleic Acids Res. 41, 1551–1563. doi: 10.1093/nar/gks1219 PMC3531112 23193283

[B28] Redelman-Sidi G. Glickman M. S. Bochner B. H. (2014). The mechanism of action of BCG therapy for bladder cancer–a current perspective. Nat. Rev. Urol. 11, 153–162. doi: 10.1038/nrurol.2014.15 24492433

[B29] Robeson M. S. 2nd O’Rourke D. R. Kaehler B. D. Ziemski M. Dillon M. R. Foster J. T.. (2021). RESCRIPt: Reproducible sequence taxonomy reference database management. PloS Comput. Biol. 17, e1009581. doi: 10.1371/journal.pcbi.1009581 34748542 PMC8601625

[B30] Saginala K. Barsouk A. Aluru J. S. Rawla P. Padala S. A. Barsouk A. (2020). Epidemiology of bladder cancer. Med. Sci. (Basel). 8, 15. doi: 10.3390/medsci8010015 32183076 PMC7151633

[B31] Shaheen W. A. Quraishi M. N. Iqbal T. H. (2022). Gut microbiome and autoimmune disorders. Clin. Exp. Immunol. 209, 161–174. doi: 10.1093/cei/uxac057 35652460 PMC9390838

[B32] Siddiqui H. Lagesen K. Nederbragt A. J. Jeansson S. L. Jakobsen K. S. (2012). Alterations of microbiota in urine from women with interstitial cystitis. BMC Microbiol. 12, 205. doi: 10.1186/1471-2180-12-205 22974186 PMC3538702

[B33] Sung H. Ferlay J. Siegel R. L. Laversanne M. Soerjomataram I. Jemal A.. (2021). Global cancer statistics 2020: GLOBOCAN estimates of incidence and mortality worldwide for 36 cancers in 185 countries. CA Cancer J. Clin. 71, 209–249. doi: 10.3322/caac.21660 33538338

[B34] Vázquez-Baeza Y. Pirrung M. Gonzalez A. Knight R. (2013). EMPeror: a tool for visualizing high-throughput microbial community data. Gigascience 2, 16. doi: 10.1186/2047-217X-2-16 24280061 PMC4076506

[B35] Weiss S. Xu Z. Z. Peddada S. Amir A. Bittinger K. Gonzalez A.. (2017). Normalization and microbial differential abundance strategies depend upon data characteristics. Microbiome 5, 27. doi: 10.1186/s40168-017-0237-y 28253908 PMC5335496

[B36] Wickham H. (2016). ggplot2: Elegant Graphics for Data Analysis (New York, USA: Springer-Verlag New York). Available at: https://ggplot2.tidyverse.org. ISBN 978-3-319-24277-4.

[B37] Wickham H. Averick M. Bryan J. Chang W. McGowan L. D. François R.. (2019). Welcome to the tidyverse. J. Open Source Software 4, 1686. doi: 10.21105/joss.01686

[B38] Yilmaz P. Wegener Parfrey L. Yarza P. Gerken J. Pruesse E. Quast C.. (2014). The SILVA and “All-species Living Tree Project (LTP)” taxonomic frameworks. Nucleic Acids Res. 42, D643–D648. doi: 10.1093/nar/gkt1209 24293649 PMC3965112

[B39] Yuasa K. Mekata T. Kiryu I. Nomura K. Sudo R. Satomi M. (2023). Aureispira Anguillae sp. nov., isolated from Japanese eel Anguilla japonica leptocephali. Arch. Microbiol. 206, 47. doi: 10.1007/s00203-023-03771-x 38160217

[B40] Zhou H. He K. Chen J. Zhang X. (2022). LinDA: linear models for differential abundance analysis of microbiome compositional data. Genome Biol. 23, 95. doi: 10.1186/s13059-022-02655-5 35421994 PMC9012043

