# Supplementary material for: Urinary microbiota changes among NMIBC patients during BCG therapy: comparing BCG responders and non-responders
Source: Front Cell Infect Microbiol. 2025 Mar 10;15:1479795. doi: 10.3389/fcimb.2025.1479795 (PMC11931020; doi:10.3389/fcimb.2025.1479795)
Supplement: Supplementary file 1 [file DataSheet1.docx]

Supplementary Material

Urinary microbiota changes among NMIBC patients during BCG therapy: Comparing BCG responders and non-responders

Toni Boban^1^, Blanka Milić Roje^2^, Dora Knezović^2^, Ana Jerončić^3^, Hrvoje Šošić^1^,Marijan Šitum^1^, Janoš Terzić^2^*

^1^Department of Urology, University Hospital of Split, Split, Croatia

^2^ Laboratory for Cancer Research, University of Split School of Medicine, Split, Croatia

^3^Department of Research in Biomedicine and Health, University of Split School of Medicine, Split, Croatia

*** Correspondence:**Corresponding Author

Prof. Janoš Terzić, MD, PhD
[janos.terzic@mefst.hr](mailto:janos.terzic@mefst.hr)


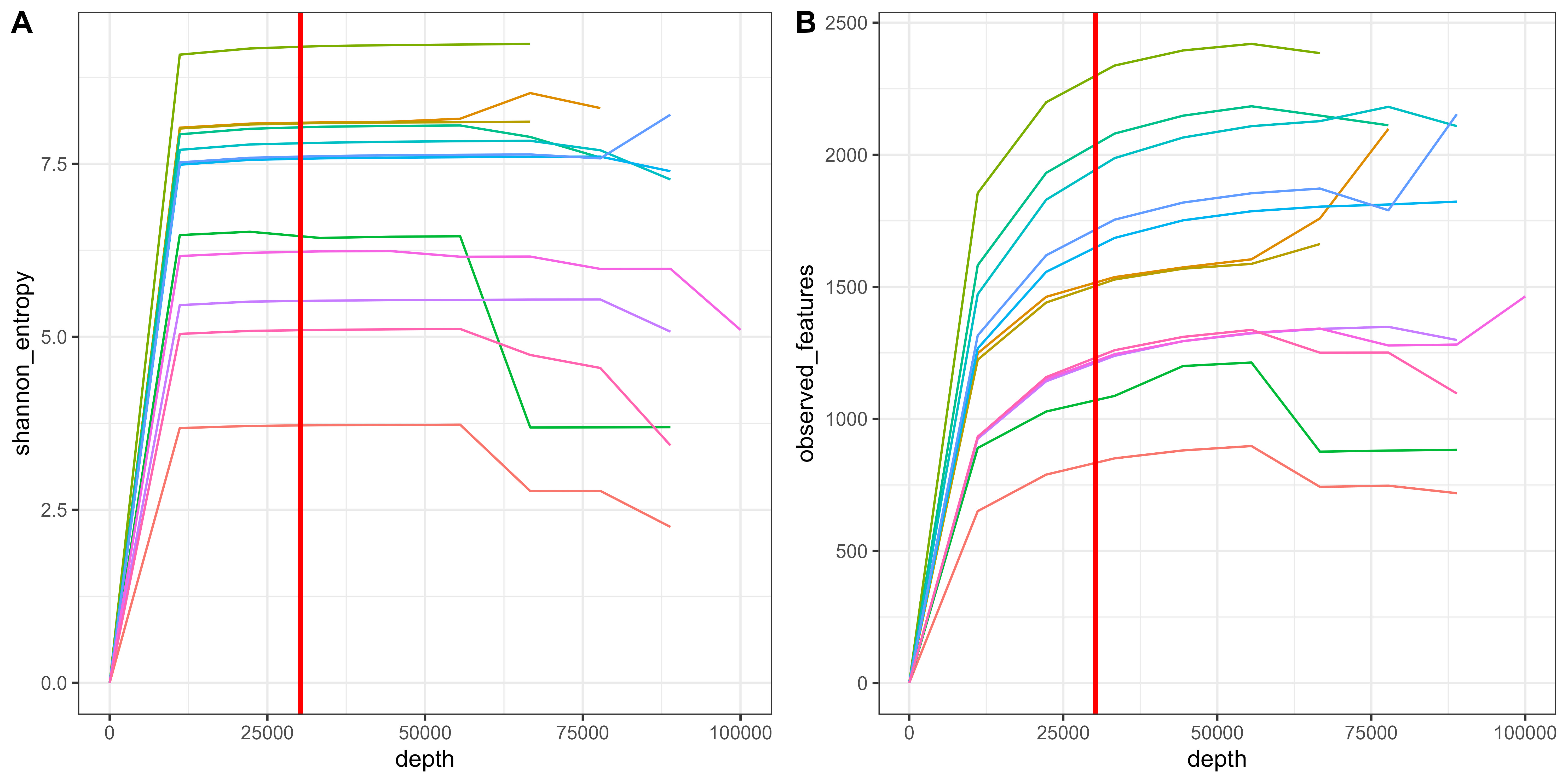


**Supplementary Figure 1.** Alpha rarefaction curves showing Shannon Index (A) and number of observed features (ASVs) (B) for the selected sequencing depth. Red line represents the selected depth for the calculation of alpha and beta diversity measures.


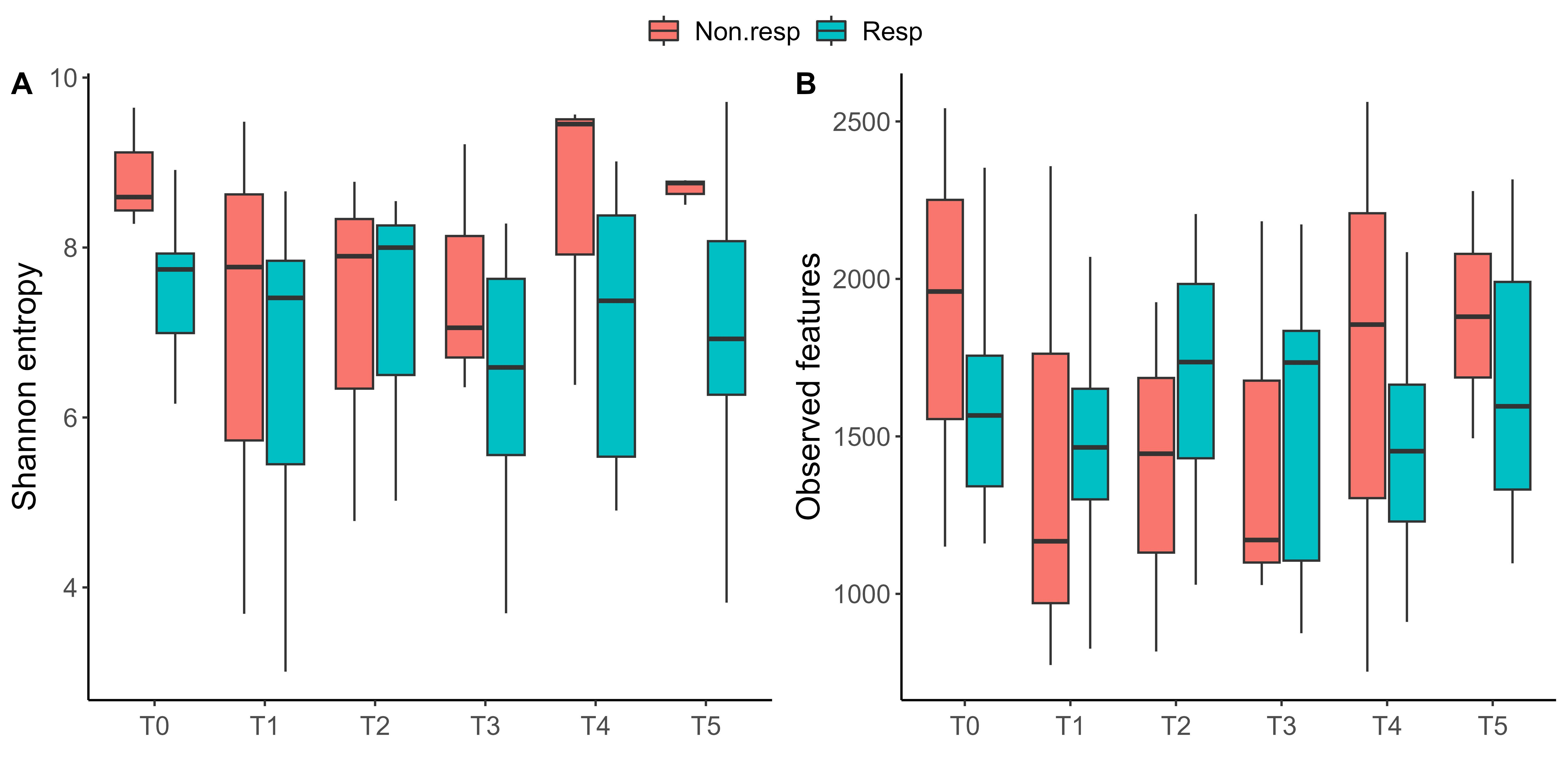
**Supplementary Figure 2.** **-** Shannon Index (entropy) (A) and number of observed bacterial features (B) in each timepoint per response-to-therapy group.


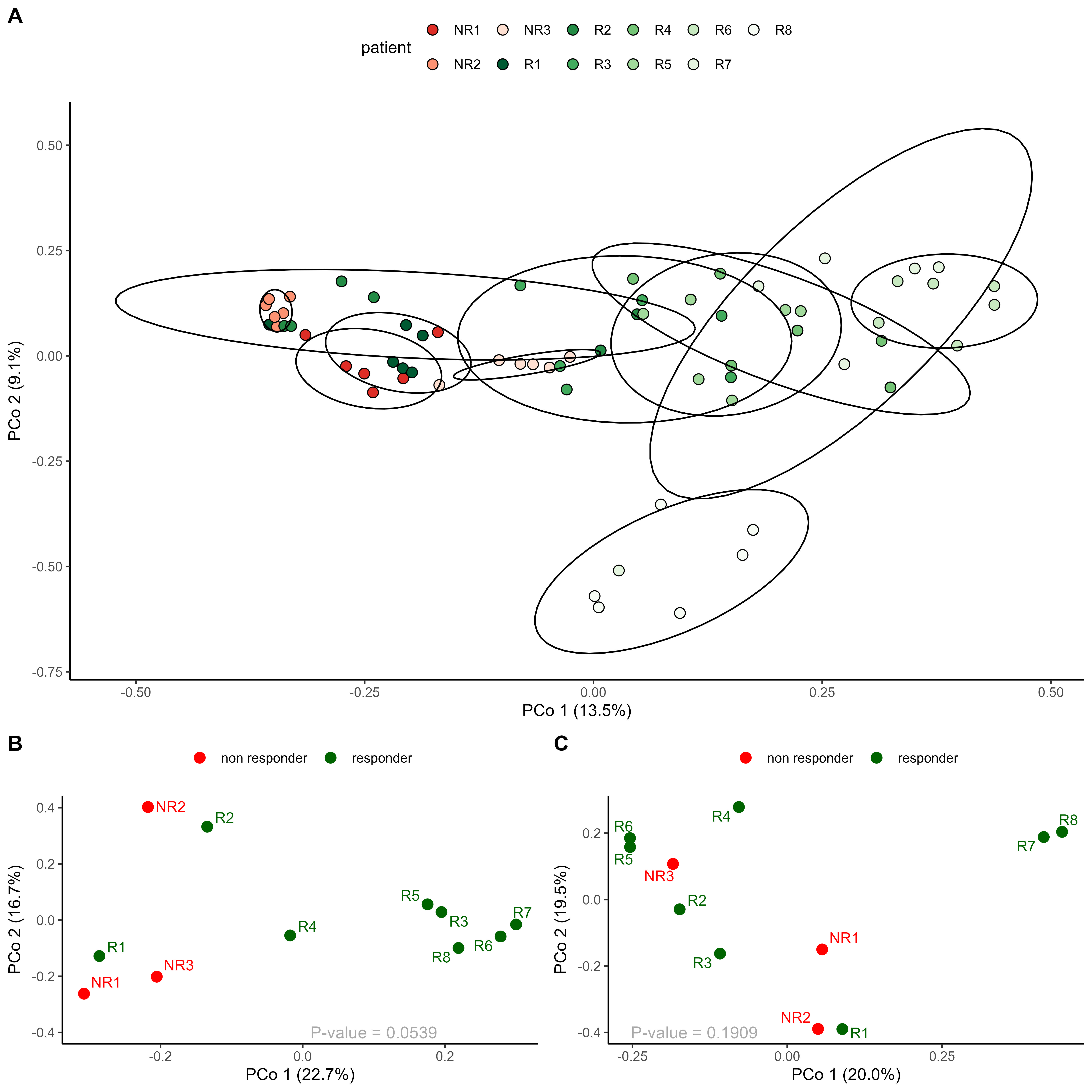
**Supplementary Figure 3.** PCoA plot of Bray-Curtis dissimilarity matrix (A) All samples grouped by patient. (B) Samples from initial timepoint (T0). (C) Samples from last timepoint (T5). P-values shown in figures A and B obtained with Permanova test with 9999 permutations.


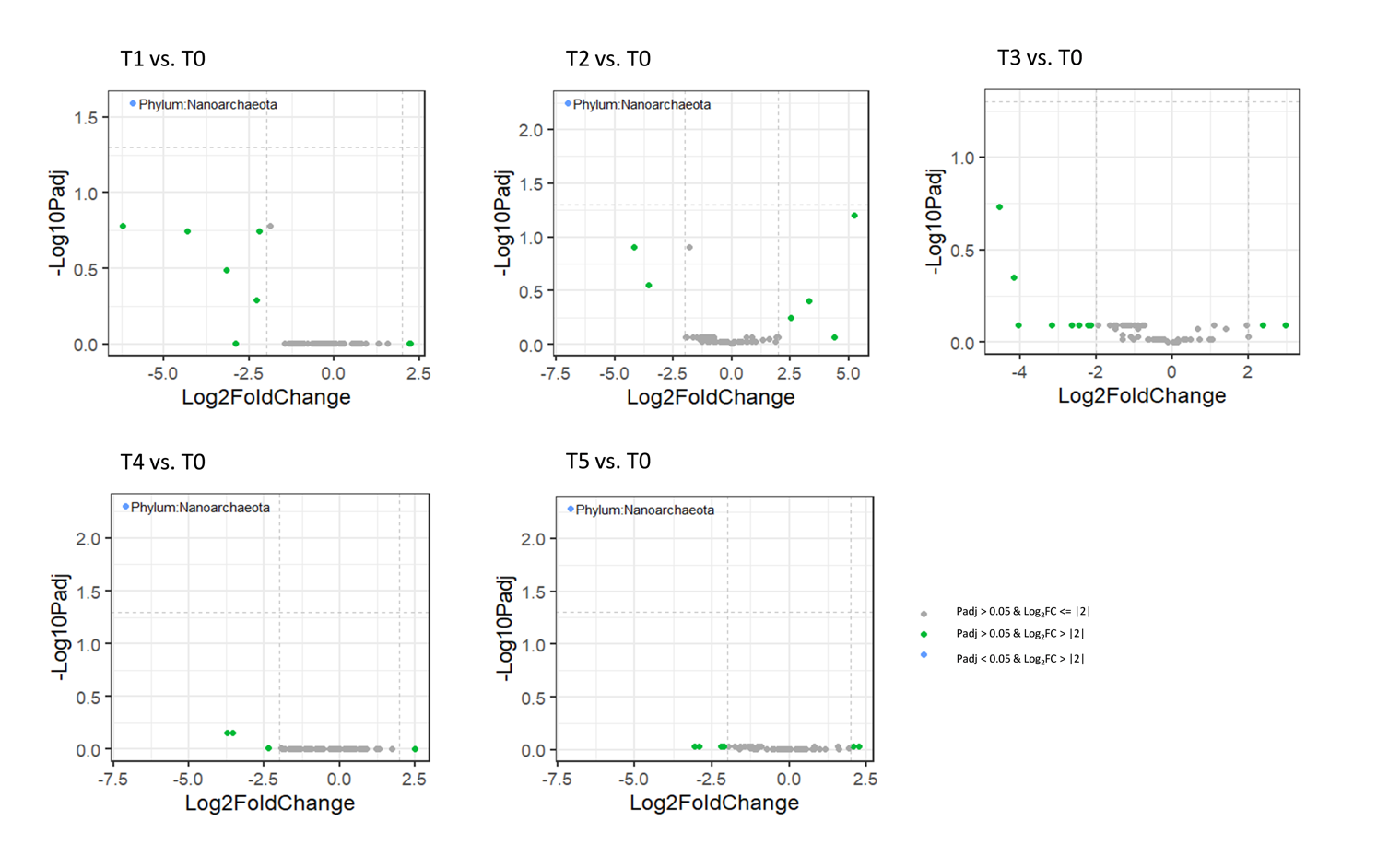


**Supplementary Figure 4.** Differential abundance analysis of urine samples between therapy timepoints in patients who did not respond to therapy (nonresponders) on phylum level (Linda analysis). The values shown in the figure are expressed as the logarithm base 2 of the fold change value (Log_2_FC). The results are represented as either positive or negative, depending on whether there is an increase or decrease in the abundance, respectively, relative to the starting point before the onset of the therapy (T0). Blue dots represent statistically significant changes in the abundance. Padj – adjusted p – value.


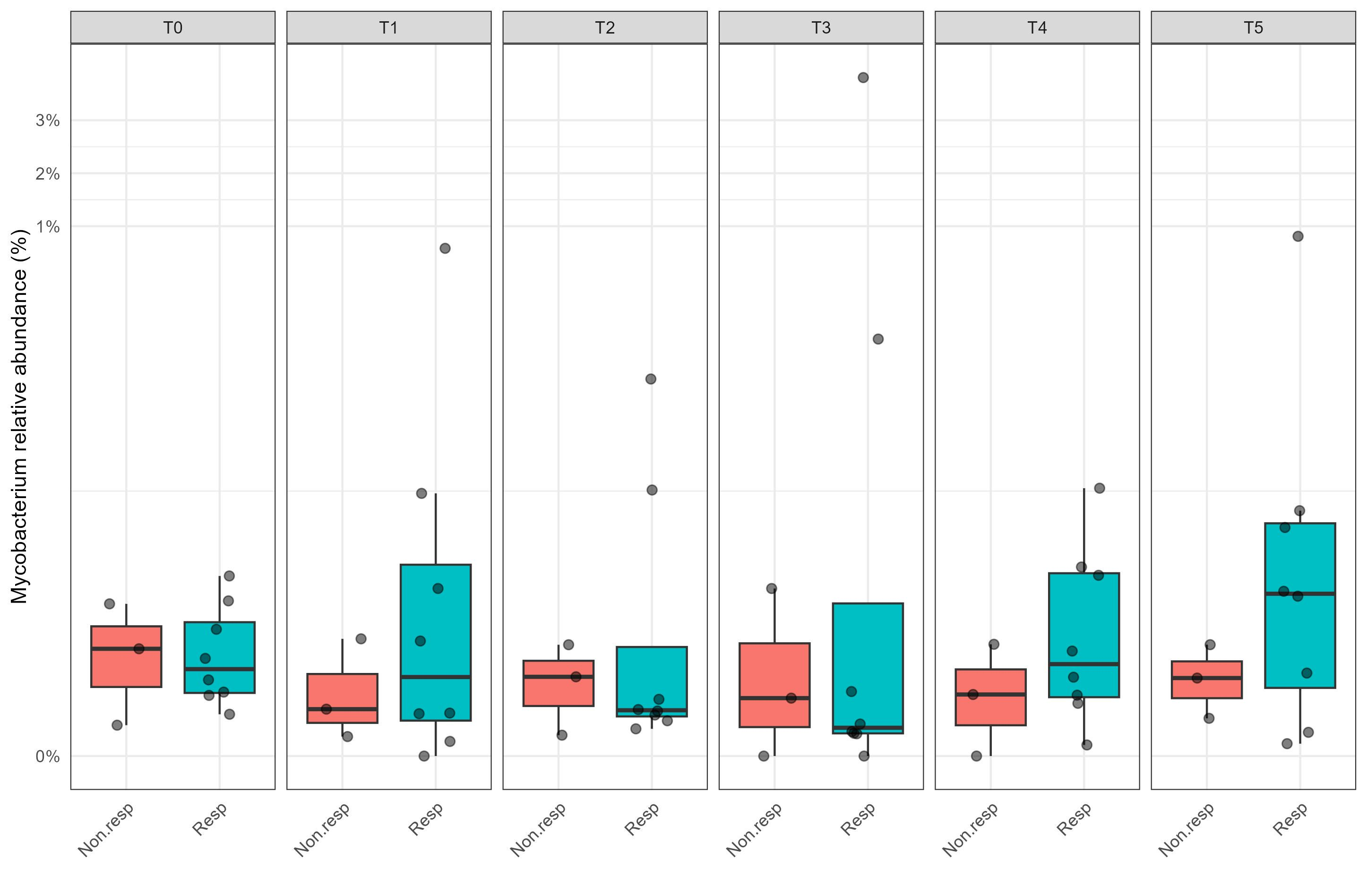
**Supplementary Figure 5.** Relative abundance the genus *Mycobacteruim*
